# The Effect of Seasonal Changes in Non-Structural Carbohydrates in Pasture on the Metabolic Profile of Horses with Laminitis

**DOI:** 10.3390/ani16020267

**Published:** 2026-01-15

**Authors:** Eva Mlyneková, Stanislav Zaťko, Marko Halo, Ivan Imrich, Marko Halo

**Affiliations:** 1 Institute of Animal Husbandry, Slovak University of Agriculture in Nitra, Tr. A. Hlinku 2, 94976 Nitra, Slovakia; eva.mlynekova@uniag.sk (E.M.); marko.halo@uniag.sk (M.H.); ivan.imrich@uniag.sk (I.I.); 2 Institute of Applied Biology, Slovak University of Agriculture in Nitra, Tr. A. Hlinku 2, 94976 Nitra, Slovakia; marko.halo1@uniag.sk

**Keywords:** fructosamines, glucose, insulin, horse, laminitis, pasture

## Abstract

This study aimed to examine the seasonal dynamics of non-structural carbohydrates in pasture and their relationship to metabolic indicators in horses with a history of laminitis. Thirty Hucul mares were divided into a laminitis group (LG, *n* = 15) and a control group (CG, *n* = 15). Insulin, glucose, fructosamines concentrations and body weight were monitored during four sampling periods (S0–S3), while pasture variables were analyzed during three periods (S1–S3). The concentration of water-soluble carbohydrates was highest in May (126.8 g/kg DM) and measured lower in October (57.9 g/kg DM), while starch concentrations measured 0.1 g/kg DM in May, 25.8 g/kg DM in July, and 24.0 g/kg DM in October. No significant differences were observed in insulin concentrations between groups (*p* > 0.05). Glucose was significantly higher in LG in May (LG: 5.50 mmol/L; CG: 5.09 mmol/L; *p* < 0.05) and October (LG: 5.98 mmol/L; CG: 5.24 mmol/L; *p* < 0.01). Fructosamine values were higher in LG throughout the season, with significance in October (LG: 120.6 μmol/L; CG: 101.1 μmol/L; *p* < 0.05). Body weight increased in both LG and CG during grazing (S2), with mean values at S0 being 423.6 kg in LG and 424.8 kg in CG, and at S2 being 533.8 kg in LG and 535.6 kg in CG (*p* > 0.05 for between-group differences). These findings suggest a different glycemic response in laminitic horses in relation to WSC and starch concentrations and highlight starch as a potential predictor of glycemic instability.

## Simple Summary

Laminitis represents a serious equine disease that results in severe pain, locomotor disorders, and, in advanced cases, irreversible structural damage within the hoof structures, leading to reduced performance and, in severe cases, possibly to the euthanasia of the affected animal. One of the most significant predisposing factors of laminitis is nutrition. The content of non-structural carbohydrates in pasture plants undergoes considerable seasonal variations depending on temperature, light conditions, humidity, and the growth stage of plants. These fluctuations can significantly affect the glycemic response and metabolic stability of animals, particularly in obese horses with limited physical activity. Examining the interrelated factors is essential for understanding the mechanisms of laminitis development under real management conditions. A comprehensive approach provides a basis for identifying critical periods of increased risk and thus enables the formulation of preventive measures. In principle, ensuring preventive measures is much more effective than the subsequent investment required to deal with the consequences of this disease.

## 1. Introduction

Pasture represents the most natural source of nutrient intake for horses. In addition to its nutritional benefits, it contributes to better body conditions, optimal constitution and improved metabolism [1]. Depending on the geographical area, properly managed pastures can represent a stable and economically advantageous source of feed that is able to cover the nutritional requirements of the horse throughout the entire grazing season [2]. Despite these benefits, pasture may not always be safe. Insufficient adaptation of the organism to fresh pasture [3] in combination with excessive intake of non-structural carbohydrates (NSCs) may lead to metabolic overload and subsequent development of laminitis [4].

According to Thorringer [5], NSCs are divided into three main groups, with starch and water-soluble carbohydrates (WSCs) representing the greatest nutritional risk. These were identified by Harris et al. [6] as the key nutritional factors involved in the development of laminitis. They also point out that most cases of laminitis occur in horses with unrestricted access to pasture. According to Hinckley and Henderson [7] up to 61% of affected animals were kept on pasture before the clinical signs appeared. The risk of laminitis on pasture does not lie only in environmental conditions and high NSC content. Hormonal disorders are also an important factor [8]. According to Lane et al. [9], an underlying endocrinopathy was present in the majority of equids with laminitis.

Currently, insulin regulation disorder, which is typical for equine metabolic syndrome (EMS), is considered the key trigger of this form of laminitis [10], which represents a set of endocrine and metabolic abnormalities [11]. It most commonly manifests as insulin dysregulation (ID) and regional adiposity [12]. Insulin resistance (IR) associated with obesity is the result of a pro-inflammatory state originating from adipose tissue [13]. Although clear data confirming a direct link between IR and laminitis are still lacking, clinical observations suggest that hyperinsulinemia and glucose intolerance considerably increase the risk [4,14]. In obesity, there is also a gradual impairment of adipose tissue function, which leads to an increased production of adipokines and amplification of the inflammatory response [15]. The systemic release of these inflammatory molecules leads to a disruption of insulin signaling and to the development or worsening of IR [16].

This indicates that IR, hyperinsulinemia and obesity represent significant risk factors for the development of laminitis and their presence is a reason for clinical examination, especially in horses with a history of recurrent laminitis [4].

The aim of this study was to analyze the seasonal dynamics of non-structural carbohydrates (WSCs and starch) in pasture and to evaluate their relationship to selected metabolic indicators (glucose, insulin, fructosamines and body weight) in horses. At the same time, the aim was to compare these relationships between a group of horses with a history of laminitis and a control group of healthy horses, with an emphasis on identifying possible risks associated with the development of laminitis. We hypothesized that higher WSC and starch values in pasture would be associated with higher glucose concentrations in horses with a history of laminitis compared to the control group.

## 2. Materials and Methods

### 2.1. Ethical Statement

All methods were carried out in accordance with the relevant guidelines and regulations. Each horse was kept and handled in accordance with the guidelines of the European Community Directive No. 86/609/EHS regarding the safety of animals used in experiments and in accordance with the ethical guidelines of the Regulation of the Slovak Republic on animal protection RD 377/12. The experimental protocol was approved by the committee of the Slovak University of Agriculture in Nitra, Slovak Republic.

### 2.2. Biological Material

A total of thirty Hucul mares were included in the study. The animals were divided into two groups based on clinical examination, anamnesis data, and the evaluation of radiographic images of the distal part of the forelimbs (latero-lateral projection). At the same time, horses were selected for the groups in such a way as to ensure a balanced age structure in both groups. During the clinical examination, we evaluated the structural integrity and signs of white line separation, manifestations of pain during manipulation of the hoof, changes in posture and lameness. The LG group included horses in which disruption of the integrity of the white line, changes in posture, lameness, and sinking or rotation of the distal phalanx were detected. The CG group included horses without pathological changes. The anamnesis was compiled on the basis of available veterinary records, taking into account previous occurrences of laminitis, episodes of musculoskeletal pain, and metabolic disorders. The radiographic examination served to assess the position of the distal phalanx relative to the hoof wall and to identify possible rotation or sinking. The LG group (*n* = 15) consisted of horses with confirmed laminitis, while the control group CG (*n* = 15) consisted of horses without signs of laminitis and without diagnosed endocrinopathy.

### 2.3. Experimental Conditions

The experiment was carried out in central Slovakia in a foothill area at an altitude of 927 m a.s.l. The horses were housed outdoors with free movement and constant access to drinking water. The facility was designed with an emphasis on safety, ethology, and social interaction between the animals. Throughout the entire duration of the experiment, the horses were kept in accordance with the recommended welfare standards for horses defined by the International Equestrian Federation (FEI Code of Conduct for the Welfare of the Horse, 2025) [17]. Feeding was based exclusively on pasture, without the supplementary provision of hay or concentrate feeds. The overall management of the housing conditions complied with the principles of good animal welfare and ensured standardized conditions for the course of the experiment.

### 2.4. Sample Collection

Sample collection was carried out from March to October 2024 in four sampling periods at 8-week intervals. Before the start of the grazing season, horses had access to hay ad libitum, without feeding on concentrate feeds. The initial sampling (S0) was performed in March before the grazing season and included blood collection only. The S1–S3 samplings (May, July, October) included parallel collection of blood and pasture samples. Both groups of horses were located on the same pasture and pasture samples were collected manually at the most intensively grazed sites. Each sample was labelled, placed in a plastic bag and transported to an accredited laboratory. The analyzed parameters were water-soluble carbohydrates (WSCs) and starch. The chemical analysis of pasture was carried out in an accredited laboratory according to the methodology for determining nutritional parameters of feed as proposed by ČSN 46 7092, part 22, in 1998 and by the Bulletin of the Ministry of Agriculture of the Czech Republic (1997), No. 2, in 1997. The analyses were performed according to the currently valid procedures of ÚKZÚZ (Czech Republic). The nutritional parameters were expressed in grams per kilogram of dry matter (g/kg).

Blood was collected from the vena jugularis (10 mL) into collection tubes (Hemos H-02) by a veterinarian in the presence of the owner. The sampling was carried out in the morning under uniform environmental and handling conditions at each sampling date. After collection, the samples were transported at 4 °C to the laboratory of the Slovak University of Agriculture, where the blood was centrifuged using a Hettich MIKRO 220R centrifuge (Andreas Hettich GmbH & Co., Tuttlingen, Germany) for 20 min at 3000 rpm, corresponding to 875× *g* when using the 1195-A rotor (24 × 0.2–2.0 mL). After centrifugation, the serum was stored at −20 °C until biochemical analyses were performed.

The determination of glucose (mmol/L) and fructosamines (µmol/L) was carried out using commercial Randox RX Monza (Randox Laboratories, Crumlin, UK) on the automatic analyzer Randox RX Monaco. The concentration of insulin (µIU/mL) was determined by the ELISA method using the Equine Insulin ELISA Kit (Mercodia Equine Insulin ELISA, Uppsala, Sweden), including the standardized procedure for incubation, washing and spectrophotometric reading. Subsequently, the calculation was performed based on the standard calibration curve. The body weight of the horses was determined using a veterinary scale, model SBS-BW-3T from the manufacturer EXPONDO POLSKA (Zielona Góra, Poland).

### 2.5. Statistical Analyses

The statistical processing of the data was carried out using the SAS Enterprise Guide software 9.4 (SAS Institute Inc., Cary, NC, USA). Methods of summary statistics, correlation and regression analyses were applied. Differences between the LG and CG groups were evaluated using a two-sample t-test. To assess changes between individual sampling periods, one-way ANOVA was used with contrast testing performed using Tukey’s test. Before the application of parametric methods, the normality of the distribution of the monitored variables was verified using Q-Q plots, histograms and normality tests. In cases where deviations from normality were identified, transformed values (logarithmic transformation) were used. In the case of correlation analyses involving non-normally distributed variables, Spearman’s correlation coefficient was applied as an alternative non-parametric approach. Values of *p* < 0.05 were considered statistically significant.

## 3. Results

As shown in Table 1, the nutritional composition of the pasture changed markedly during the grazing season. The highest concentration of WSC was recorded in May, reaching 126.80 g/kg DM. In the following months, WSC values were lower. In July, the WSC value was 90.90 g/kg and in October 57.90 g/kg DM. In the case of starch, values differed between sampling periods. The lowest concentration of starch was recorded in May (S1) at 0.10 g/kg DM. In July (S2), the measured concentration was 25.80 g/kg, and in October (S3), it was 24.00 g/kg DM.

**Figure 1:**
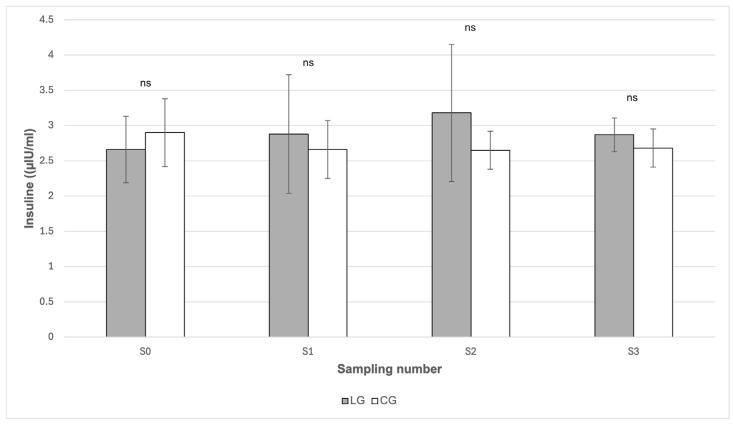
Comparison of serum insulin concentration between the LG and CG groups in individual sampling periods. LG—Laminitis group (horses with laminitis), CG—Control group (horses without laminitis), Sampling periods S0 (March), S1 (May), S2 (July), S3 (October). ns *p* > 0.05.

**Figure 2:**
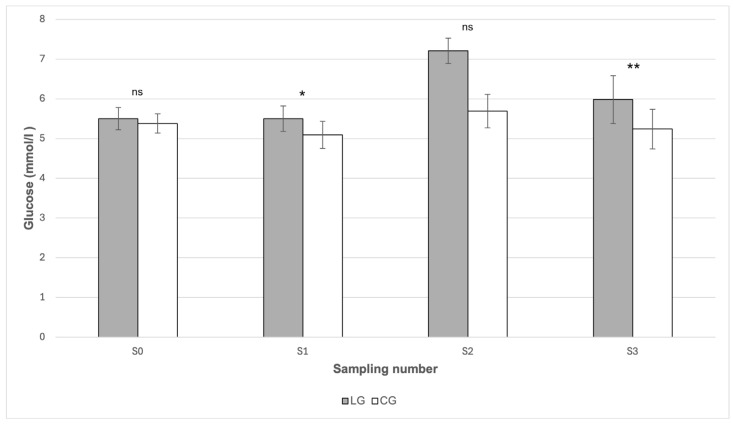
Comparison of serum glucose concentration between the LG and CG groups in individual sampling periods. LG—Laminitis group (horses with laminitis), CG—Control group (horses without laminitis), Sampling periods S0 (March), S1 (May), S2 (July), S3 (October). ns *p* > 0.05, * *p* < 0.05, ** *p* < 0.01.

From Figure 1, it can be seen that the insulin values did not show any statistically significant differences between the groups in any of the sampling periods (*p* > 0.05). In the case of glucose (Figure 2), statistically significant differences between the groups were found in May (S1) and in October (S3). In May (S1), the mean glucose value was statistically significantly (*p* < 0.05) higher in the LG group (5.50 mmol/L) compared to the CG group (5.09 mmol/L). Likewise, in October (S3) the glucose value was significantly higher (*p* < 0.01) in LG (5.98 mmol/L) than in CG (5.24 mmol/L). From Table 1, it follows that the highest mean glucose values were recorded in both groups during July (S2). The value in LG reached 7.21 mmol/L; however, when compared with CG (5.69 mmol/L), no statistically significant difference was proven (*p* > 0.05) (Figure 2). From Table 1, it can also be seen that it was precisely in this period that the concentration of starch in the pasture reached its maximum values (25.80 g/kg).

**Figure 3:**
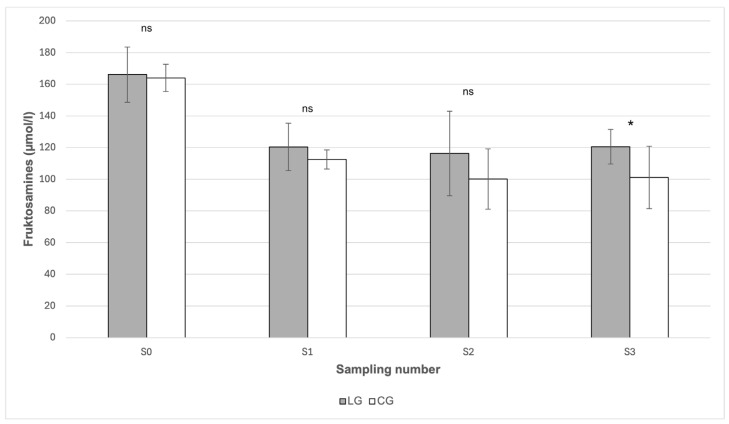
Comparison of serum fructosamine concentration between the LG and CG groups in individual sampling periods. LG—Laminitis group (horses with laminitis), CG—Control group (horses without laminitis), Sampling periods S0 (March), S1 (May), S2 (July), S3 (October). ns *p* > 0.05, * *p* < 0.05.

The highest mean value of fructosamine (166.09 μmol/L) was recorded before the start of the grazing season in March (S0) in the LG group (Table 1), however, in comparison with the value in the CG group (164.02 μmol/L), no statistically significant difference between these values was demonstrated (*p* > 0.05). The only significant difference between the groups (*p* < 0.05) occurred in October (S3), when the measured concentration of fructosamine in LG (120.58 μmol/L) was higher compared to the value of 101.1 μmol/L in CG (Figure 3).

**Figure 4:**
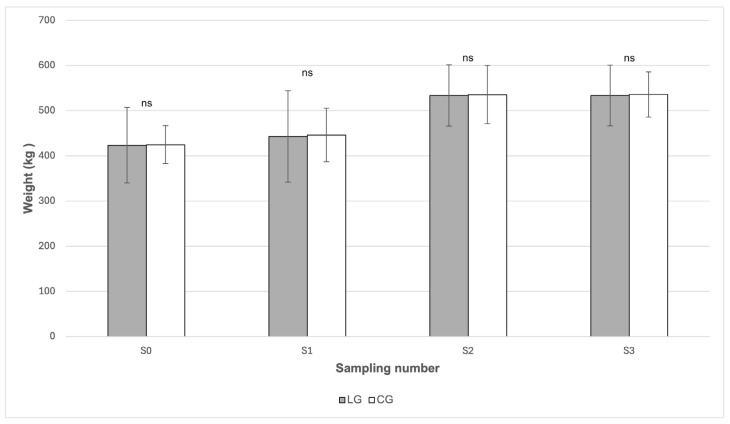
Comparison of body weight of horses between the LG and CG groups in individual sampling periods. LG—Laminitis group (horses with laminitis), CG—Control group (horses without laminitis), Sampling periods S0 (March), S1 (May), S2 (July), S3 (October). ns *p* > 0.05.

From Figure 4 it is evident that no statistically significant differences in body weight between the LG and CG groups were demonstrated in any of the sampling periods (*p* > 0.05). In both groups, body weight showed a similar development during the grazing season. At the beginning of the study (S0), the mean body weight was comparable between the LG group (423.60 kg) and the CG group (424.80 kg). After the onset of grazing (S1), a moderate increase in body weight was observed in both groups, reaching 443.00 kg in the LG group and 446.20 kg in the CG group. During July (S2), mean body weight increased further and reached the highest values in both groups, specifically 533.80 kg in LG and 535.60 kg in CG, with these values remaining stable until October (S3), when mean body weight reached 533.80 kg in LG and 535.80 kg in CG. According to the data presented in Table 1, changes in body weight between sampling periods were statistically significant only in the CG group (*p* = 0.0073), whereas no statistically significant changes were detected in the LG group.

**Figure 5:**
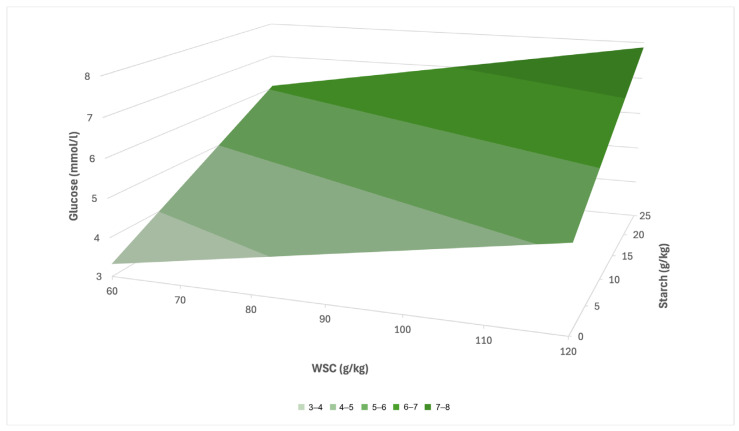
Predicted effect of WSC and starch in pasture on serum glucose concentration in LG.

Based on the results of the multiple linear regression, a prediction equation was created for the LG group in the form: y = 1.52 + 0.03x_1_ + 0.11x_2_ (R^2^ = 0.39), where the variable x_1_ represents the concentration of WSC and x_2_ the concentration of starch in the pasture (g·kg^−1^ dry matter). Based on this model, it can be assumed that with increasing values of WSC and starch in pasture, there will be a pronounced increase in serum glucose concentration (Figure 5). At low concentrations of these nutritional indicators, the estimated serum glucose level ranges around 3–4 mmol/L, while at their maximum values, it may reach up to 7–8 mmol/L. A more pronounced effect can be expected in the case of starch, which, according to the model, contributes to a higher increase in glucose than WSC.

**Figure 6:**
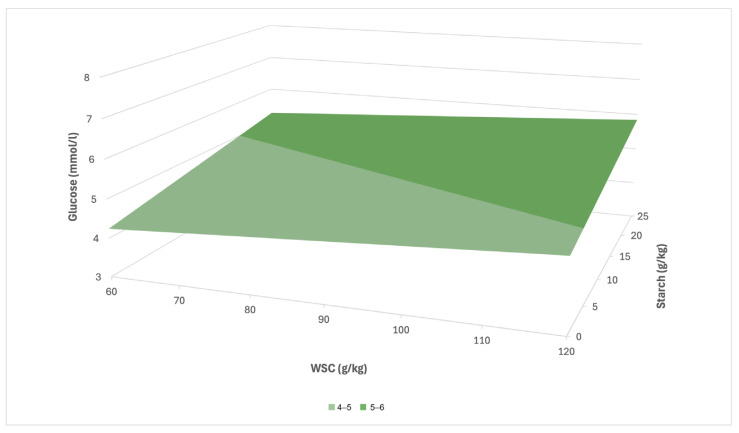
Predicted effect of WSC and starch in pasture on serum glucose concentration in CG.

In the case of CG, a prediction equation in the form y = 3.64 + 0.01x_1_ + 0.04x_2_ (R^2^ = 0.15) was created based on the multiple linear regression, where x_1_ also denotes the concentration of WSC and x_2_ the concentration of starch. In the CG group, this relationship was considerably weaker (Figure 6). At low values of WSC and starch, it can be assumed that the serum glucose concentration will remain below the threshold of 5 mmol/L, while at the maximum concentrations of both parameters, it could reach approximately 6.5–7.0 mmol/L. Compared to LG, a slower increase in glucose in CG and a weaker response to increasing nutritional input factors can therefore be expected.

**Figure 7:**
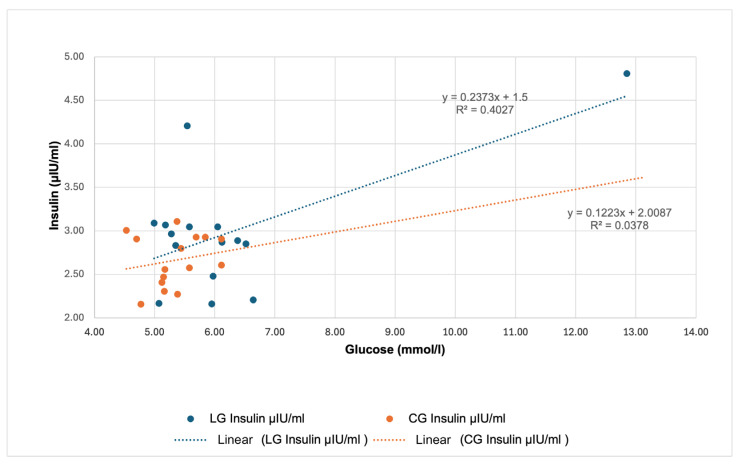
Predicted effect of glucose concentration on serum insulin concentration in horses with laminitis (LG) and without laminitis (CG) (*p* > 0.05).

Figure 7 visualizes the relationship between glucose concentrations and insulin concentrations in LG and CG. In the case of LG, the regression equation was determined as y = 0.2373x + 1.5, with the coefficient of determination R^2^ = 0.4027 indicating a moderately strong positive relationship between the two variables. With increasing glucose concentration, there is also a more pronounced increase in insulin concentrations in LG. In CG, the calculated regression equation was y = 0.1223x + 2.0087, but the R^2^ value was only 0.0378. Thus, in CG, the change in glucose concentration influences the insulin response only minimally, which is also reflected in the markedly lower slope of the trend line. These results indicate that in horses with a history of laminitis, elevated glucose concentrations may have a more substantial effect on insulin secretion.

## 4. Discussion

The seasonal dynamics of NSC content in pasture stands are a key factor influencing their nutritional value and the risk of developing metabolic disorders in horses [18]. According to [6], high concentrations of WSC as well as an increased starch content may contribute to the development of pasture-associated laminitis. Our results demonstrated a decreasing trend in WSC concentrations during the observed period, with the highest level recorded in May (126.80 g/kg dry matter) and the lowest in October (57.90 g/kg dry matter). These results correspond to known seasonal changes, in which maximum values are usually reached at the end of spring, a decline occurs during the summer and concentrations stabilize at a moderate level in the autumn months [18]. The authors further add that a similar development of WSC was observed in a study conducted on ten horse farms in Germany, where the highest values were recorded in May and the lowest in August. The October concentrations were within the medium range, which indicates a consistent seasonal decline in NSC accumulation across different climatic conditions.

While the concentration of WSC in our case showed a gradual decrease, the starch content increased markedly in July (25.80 g/kg dry matter), whereas in May it was practically zero (0.1 g/kg dry matter). In October, we observed only a slight but statistically significant decrease (*p* < 0.05) to 24.00 g/kg dry matter. Although starch forms only a minor component of the vegetative parts of temperate grasses (usually <35 g/kg dry matter), in generative structures, especially seeds, it may reach 330–440 g/kg dry matter [19]. This suggests that the botanical composition of the pasture, including the proportion of seed structures, can significantly affect the overall intake of starch. In this context, it is worth mentioning the statement of Meyer et al. [20] and Potter et al. [21], who recommend 2 to 4 g/kg body weight as the maximum safe single dose of starch, which for a 500 kg horse corresponds to 1 to 2 kg of starch per day [18]. Similarly, Vervuert et al. [22] described a statistically significant increase in serum glucose (*p* < 0.05) within 30 to 60 min after feed administration, with the highest values occurring in cases where starch intake exceeded 1.1 g/kg live weight. These findings support the assumption that even under a natural grazing regime, a significant glycemic stimulus may occur, especially when there is a sudden intake of starch.

In our analysis, starch also proved to be a significant predictor of glucose level, especially in horses in LG (WSC = 0.03, starch = 0.11), which indicates its role in increasing glycaemia (Figure 5). In the regression model, it was shown that the combination of high concentrations of WSC (120–130 g/kg) and starch (25–26 g/kg) corresponds to the highest predicted glucose concentrations (7–9 mmol/L). In CG, the effect of nutritional parameters on glucose was considerably weaker (WSC = 0.01, starch = 0.04). In addition, the difference in mean glucose concentrations between the LG and CG groups was shown to be statistically significant in several sampling periods (S1 *p* < 0.05; S2 *p* < 0.01), which indicates the presence of metabolic dysregulation in horses with a history of laminitis. In contrast, in the study by Knowles et al. [23], increased glucose values were recorded in only two individuals, which the authors reported as isolated cases without a statistically significant difference (*p* > 0.05) between the monitored groups.

Although according to Siciliano et al. [24] the exact mechanism of the development of pasture-associated laminitis is not known, one possibility is that increased NSC intake raises the flow of glucose into the bloodstream, which leads to an intensified insulin response. In this context, it is particularly important to note one case of clinically significant hyperglycemia, which in our experiment occurred precisely during the period of the highest starch load (25.80 g/kg). In one individual from the LG group, a glucose level of 12.85 mmol/L was recorded in July, which markedly exceeded the reference interval for healthy horses (4.2–7.3 mmol/L; [25]). This condition was not accompanied by an increased insulin level (4.81 µIU/mL), which may indicate impaired insulin sensitivity in this individual. Approximately three weeks after this sampling, clinical signs of laminitis appeared in the monitored mare, which creates a temporal association between increased starch intake, hyperglycemia, and the onset of the disease.

Most of the glucose values measured in our study were within the physiological range, with the mean values of horses with laminitis often being closer to the upper limit of the reference interval. Statistically significantly higher glucose values in LG compared to CG were recorded in May (LG: 5.50 mmol/L; CG: 5.09 mmol/L; *p* < 0.05) and in October (5.98 mmol/L; 5.24 mmol/L; *p* < 0.01). In July, although the mean glucose level in LG was the highest (7.21 mmol/L), the difference compared to CG (5.69 mmol/L) did not reach statistical significance (*p* > 0.05). These results may indicate reduced glycemic stability in horses with a history of laminitis. The glucose values we measured correspond to the findings of Frank et al. [11], who state that hyperglycemia in horses with EMS is rarely detected, because most animals maintain an effective compensatory insulin secretory response despite IR. They also add that glucose concentrations are often near the upper limit of the reference range, which indicates a partial loss of glycemic control.

Fructosamine represents a medium-term indicator of average glycaemia over a period of 2–3 weeks and may be particularly useful in situations where the current glucose level may not reflect a stable metabolic state [26]. According to Schleicher et al. [27], fructosamines arise through non-enzymatic glycation of plasma proteins, mainly albumin. In this context, they provide an important supplementary parameter in the assessment of the glycemic profile. The concentrations of fructosamine were higher in LG than in CG throughout the observed period, but a statistically significant difference was demonstrated only in October (*p* < 0.05), when the concentration in LG was 120.58 μmol/L compared to 101.1 μmol/L in CG. This indicates a slightly increased chronic glycaemia in horses with laminitis, particularly in the later phase of the grazing season. However, it should be noted that all values were well below the reference limit of 248.7 μmol/L [28].

In contrast to our findings, where the difference between the groups was not consistently statistically significant, Knowles et al. [23] recorded significantly higher fructosamine concentrations (*p* < 0.001) in horses with laminitis, which suggests that changes in concentrations may also be associated with the phase of the disease or its acuteness. From Table 1, it can be seen that a statistically significant decrease in fructosamine concentration occurred after the S0 sampling in both groups. Since this sampling was carried out before the transition to controlled grazing conditions, one possible explanation may be that the change in diet played an important role in the adjustment of the glycemic profile. However, according to Murphy et al. [29], the interpretation of fructosamine values may also be influenced by the level of serum proteins, especially albumin, which shows a reduced concentration as a response to the presence of inflammation [30].

The presence of inflammation could be explained by changes in the average body weight of the horses during the grazing season. In both groups, an increase in body weight was recorded during grazing (S2) (Table 1), which temporally coincided with a higher content of energetically relevant pasture components. According to the data presented in Table 1, the concentration of WSC reached 126.80 g/kg dry matter at the beginning of the grazing season (S1), and the starch content increased markedly in July (S2) to 25.80 g/kg dry matter, which may have led to a positive energy balance and a subsequent increase in body weight. Obesity directly affects increased circulating concentrations of pro-inflammatory cytokines [31] and since increased systemic inflammation is a common risk factor for the development of laminitis, it is possible that inflammation associated with obesity is the aspect that predisposes horses with EMS as at risk [32]. Moreover, obesity is also associated with increased plasma insulin concentrations [32,33], which was not confirmed in our case. None of the measured values exceeded the threshold of 20 μIU/mL, which is given by Johnson et al. [34] as the upper physiological limit. Furthermore, we did not observe statistically significant differences in insulin concentrations between LG and CG. In contrast to our results, Knowles et al. [23] report the presence of fasting-induced hyperinsulinemia in up to 43% of the monitored horses, which may indicate some methodological differences in the experiment, including the timing of sampling, the type of analysis used and nutritional management prior to testing. The conditions of sample collection are important in the diagnosis of chronic IR associated with EMS, since cortisol and adrenaline released as a result of pain or stress decrease tissue sensitivity to insulin and increase resting concentrations of glucose and insulin [35,36]. It also remains debatable whether differences exist in the mechanisms by which starch and WSC contribute to the development of laminitis, since both types of NSCs may lead to an increased insulin response, particularly in horses with IR [37].

## 5. Conclusions

The results of our study indicate a pronounced seasonal variability in the content of WSC and starch in pasture, which proved to be significant predictors of glycaemia in horses. While in the group of horses with laminitis the effect of nutritional factors on blood glucose concentration was more pronounced, the control group showed a more stable metabolic profile. The presence of statistically significantly higher glucose values in LG during certain periods confirms the reduced glycemic stability of these horses. Insulin concentrations did not differ significantly between the groups and remained below the reference limit. The findings obtained highlight the importance of seasonal monitoring of pasture, an individual approach to the management of at-risk horses, and the need to monitor not only current glycaemia, but also medium-term indicators such as fructosamines and body weight. The early identification of risk factors and appropriate dietary adjustments may contribute significantly to the prevention of pasture-associated laminitis.

## Figures and Tables

**Table 1:** Mean values of metabolic and nutritional indicators in LG and CG.

	Parameter	S0	S1	S2	S3	SEM	*p*-Value
LG	Insulin (µIU/mL)	2.66	2.88	3.18	2.87	0.08	ns
	Glucose (mmol/L)	5.50	5.50	7.21	5.98	0.09	ns
	Fructosamines (µmol/L)	166.09 ^a^	120.41 ^b^	116.27 ^b^	120.58 ^b^	6.72	0.0016
	Weight (kg)	423.60	443.00	533.80	533.80	20.33	ns
CG	Insulin (µIU/mL)	2.90	2.66	2.65	2.68	0.15	ns
	Glucose (mmol/L)	5.38	5.09	5.69	5.24	0.37	ns
	Fructosamines (µmol/L)	164.02 ^a^	112.48 ^b^	100.07 ^b^	101.10 ^b^	6.02	<0.0001
	Weight (kg)	424.80 ^a^	446.20 ^ab^	535.60 ^b^	535.80 ^b^	16.15	0.0073
Pasture	WSC (g/kg)	-	126.80	90.90	57.90		
	Starch (g/kg)	-	0.10	25.80	24.00		

LG—Laminitis group (horses with laminitis), CG—Control group (horses without laminitis), WSC—Water-soluble carbohydrates, Sampling periods S0 (March), S1 (May), S2 (July), S3 (October). The indices ^a^, ^b^ in the rows indicate significant differences at the significance level *p* < 0.05, ns *p* > 0.05.

## Data Availability

Data are contained within the article.

## Abbreviations

The following abbreviations are used in this manuscript:

CG	Control group
DM	Dry matter
EMS	Equine metabolic syndrome
ID	Insulin dysregulation
IR	Insulin resistance
LG	Laminitis group
NSC	Non-structural carbohydrates
WSC	Water-soluble carbohydrates
